# Rare complications of ERCP—pancreatitis, panniculitis, and polyarthritis syndromeand multifocal osteomyelitis

**DOI:** 10.1093/bjrcr/uaaf002

**Published:** 2025-01-17

**Authors:** Ratna Koyyalamudi, Dominic Ku, Kirk Brown, Morgan Schulze

**Affiliations:** Department of Radiology, John Hunter Hospital, Newcastle 2305, Australia; Department of Radiology, John Hunter Hospital, Newcastle 2305, Australia; Department of Radiology, John Hunter Hospital, Newcastle 2305, Australia; Department of Radiology, John Hunter Hospital, Newcastle 2305, Australia

**Keywords:** pancreatitis, panniculitis, polyarthritis, ERCP

## Abstract

Severe pancreatitis following retrograde cholangiopancreatography (ERCP) is an infrequent occurrence. Even rarer are the additional non-pancreatic symptoms that can emerge after ERCP-induced pancreatitis, such as panniculitis and polyarthritis. This combination of symptoms is recognized as the pancreatitis, panniculitis, and polyarthritis syndrome (PPPS). PPPS typically manifests as reddish subcutaneous nodules, primarily in the lower extremities. In some cases, the condition may progress into the bones, causing intramedullary fat necrosis/bone infarcts. Joint complications involve polyarthritis and affect both small and large joints. PPPS tends to develop 3-6 weeks after the peak of clinical pancreatitis. In this report, we present a case of PPPS that occurred as a complication following ERCP. This particular case became further complicated by the presence of bacteraemia and fungaemia, leading to the subsequent development of multi-focal osteomyelitis. The medical imaging included in this report provides a comprehensive overview of the entire clinical course, starting from the initial post-procedural complication and pancreatitis, followed by multi-modal imaging depicting panniculitis using ultrasound and MRI, and finally illustrating the development of multi-focal osteomyelitis. This case serves as an opportunity to explore and understand 2 rare complications associated with pancreatitis: PPPS and multi-focal osteomyelitis.

## Introduction

Pancreatitis can lead to manifestations outside the pancreas, such as lobular panniculitis and polyarthritis. When these symptoms occur together, they are collectively referred to as the pancreatitis, panniculitis, and polyarthritis syndrome (PPPS).

In a case series, acute pancreatitis was identified as the primary cause in the majority of cases, accounting for approximately 60%, while chronic pancreatitis was responsible for 40% of cases.^1^ Less frequently, pancreatic tumours, including acinar cell types and pseudopapillary pancreatic tumours, were implicated as rarer contributing factors.

Panniculitis, a condition characterized by the development of erythematous subcutaneous nodules, was found to be an infrequent association, affecting up to 3% of individuals with pancreatic disease.^2^ These nodules typically appear on the lower extremities and can develop into ulcers with exudative fat necrosis.^1^ In some cases, fat necrosis may extend to the bones, resulting in intramedullary fat necrosis.

Joint involvement was observed in patients with pancreatitis, primarily manifesting as polyarthritis, which affected both small and large joints in a significant majority of cases (88%). However, a minority of cases presented with oligoarthritis or monoarthritis.^1^ The joints most commonly affected included the ankles, knees, wrists, and metacarpophalangeal joints.

According to a case series involving 25 patients, arthritic changes typically manifested 3-6 weeks after the peak of clinical pancreatitis. A transient course was slightly more common, but a substantial portion of patients (44%) experienced a chronic course with a limited response to treatment. Additionally, it is worth noting that osteomyelitis has been reported as a rare complication of PPPS.

## Case report

We share a unique case that illustrates the complexities arising from pancreatitis induced by ERCP, including a detailed examination of the initial pancreatitis symptoms, diverse imaging of panniculitis, polyarthritis, bone infarcts, and the subsequent emergence of multifocal osteomyelitis. This case report serves to increase awareness of an infrequent complication related to ERCP and pancreatitis in general, known as the pancreatitis, polyarthritis, and panniculitis syndrome.

This clinical case involved a 64-year-old patient who experienced post-ERCP and sphincterotomy-induced pancreatitis in September 2022. The ERCP was carried out for choledocholithiasis and extensive cholelithiasis; common bile duct and pancreatic duct stents were placed without documented complication. The patient sought medical attention at the emergency department just 2 days after an outpatient procedure, presenting with symptoms such as fevers, rigors, nausea, and abdominal pain. Initial lipase levels were measured at 336 U/L, later escalating to 10 000 U/L. The white cell count was 15 × 10^9^/L and the C-reactive protein was 61 mg/L.

**Figure 1.**
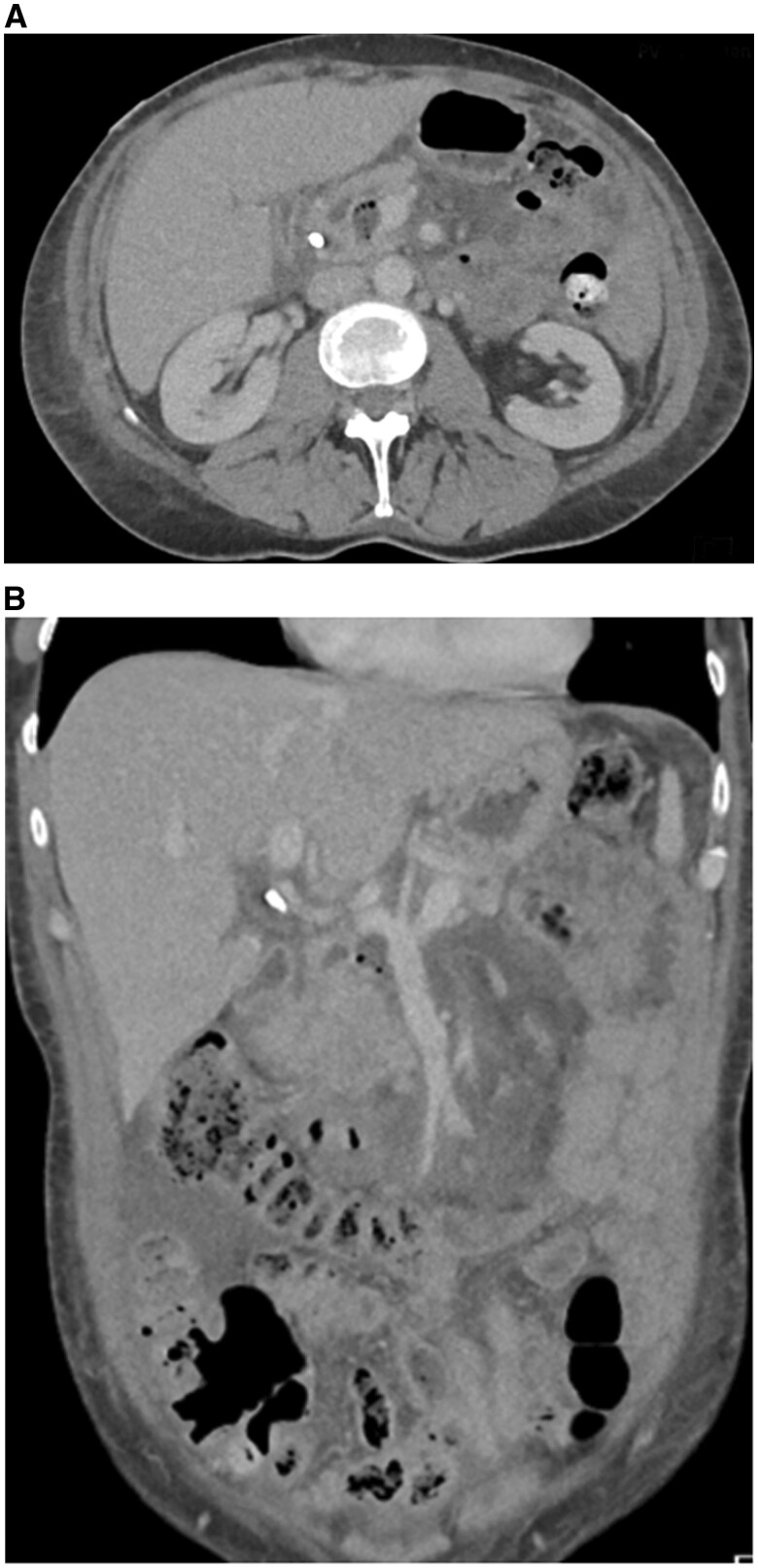
(A) Pancreatic head collection and surrounding inflammatory changes. Portal venous contrast-enhanced CT axial image of the upper abdomen demonstrates a collection at the pancreatic head containing gas locules consistent with pancreatic duct perforation. No pancreatic necrosis. Extensive inflammatory changes in peripancreatic tissues and mesentery. Common bile duct stent *in situ*. (B) Pancreatitis with surrounding inflammatory changes and free fluid. Portal venous contrast-enhanced CT coronal image demonstrates inflammatory change and free fluid centred on pancreas consistent with pancreatitis. Common bile duct stent *in situ*.

**Figure 2.**
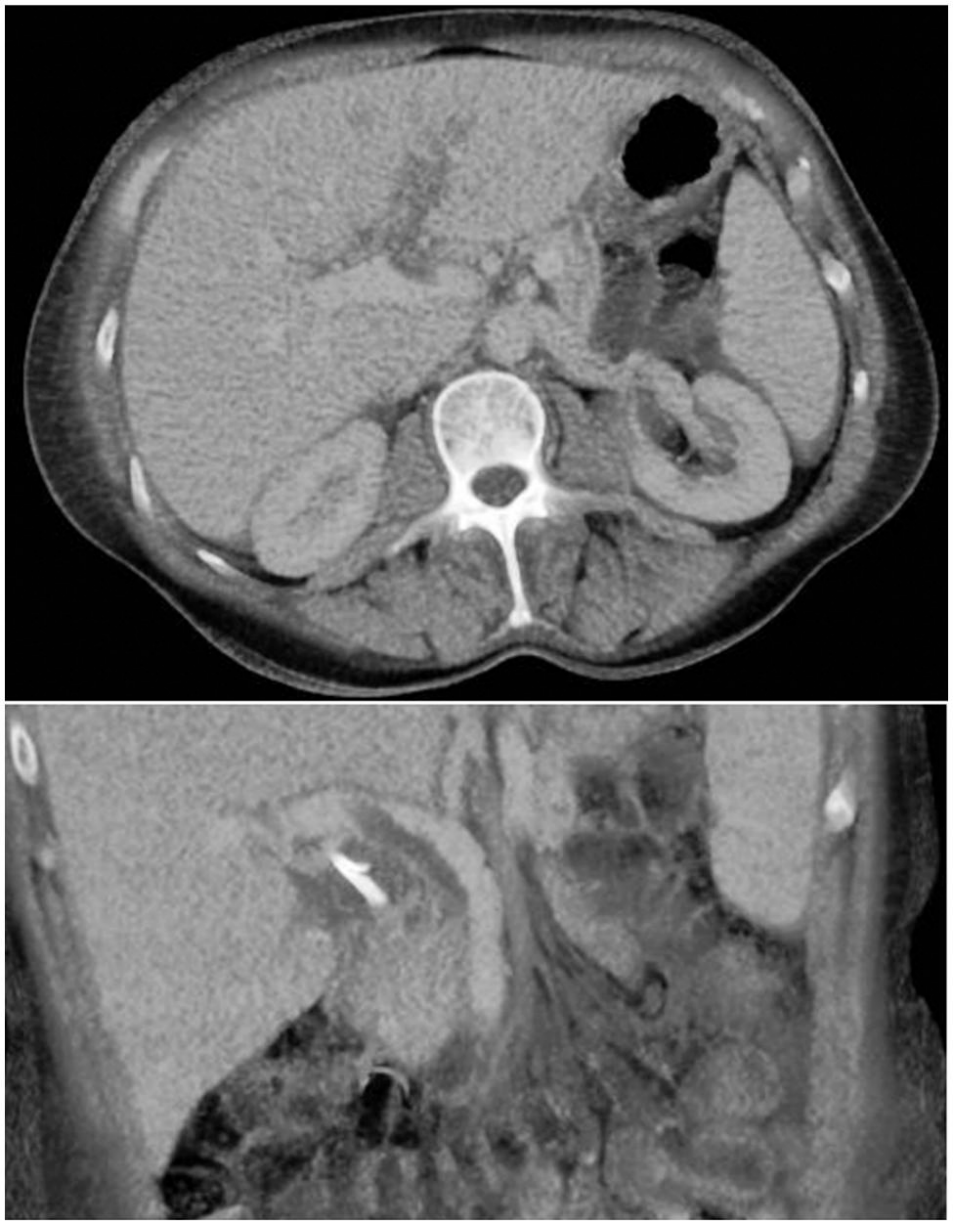
Portal vein thrombus extending into the left portal vein. Portal venous contrast-enhanced CT with maximal intensity projection (MIP) images demonstrates partially occlusive portal vein thrombus extending into left portal vein. Common bile duct and pancreatic stents are *in situ*.

An initial CT scan revealed findings consistent with pancreatitis and a pancreatic head collection resulting from a perforated pancreatic duct (Figure 1A and B). Findings were consistent with modified CT Severity Index grade D. There was no evidence of pancreatic necrosis. On a subsequent CT, further complication with the development of portal vein thrombosis was seen (Figure 2). Additionally, the patient developed bloodstream infections caused by *Klebsiella oxytoca* and *Streptococcus milleri*, along with fungaemia due to *Candida albicans*. Treatment involved the administration of intravenous Augmentin and a long-term course of fluconazole.

**Figure 3.**
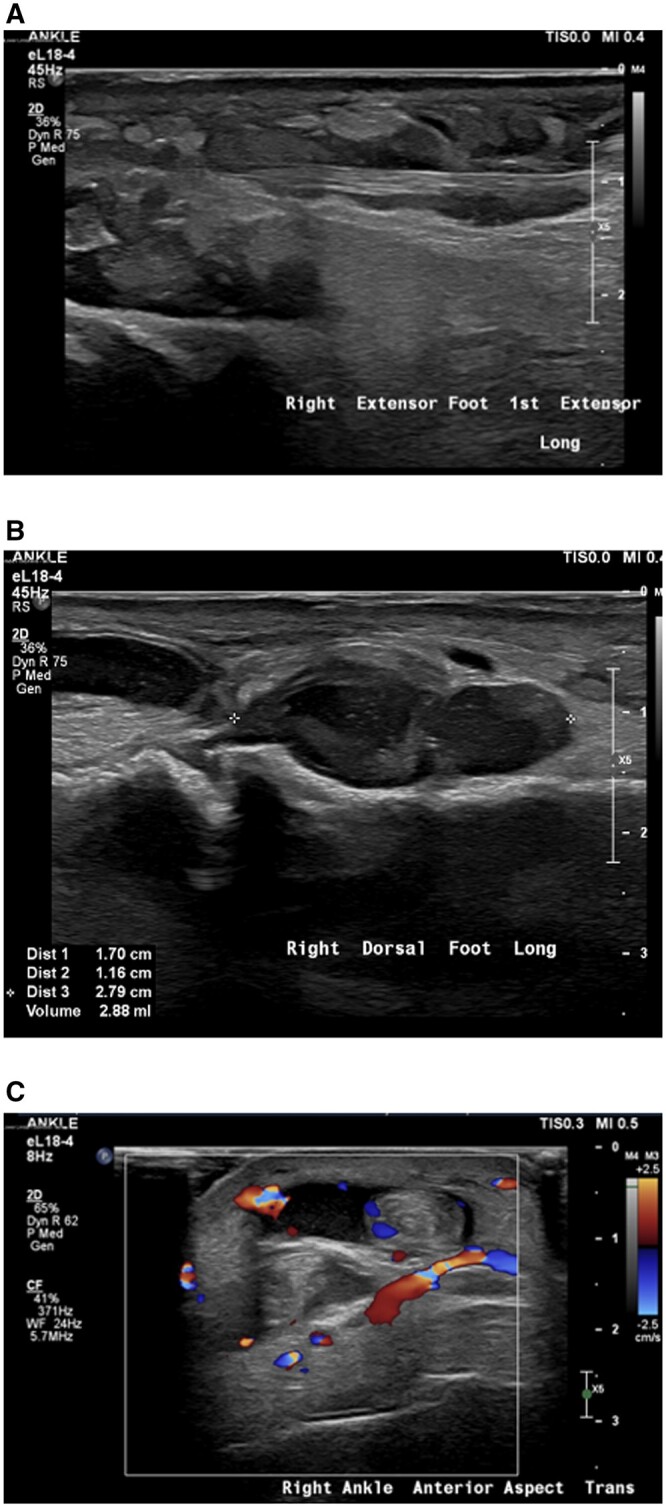
Ultrasound images demonstrate the appearance of subcutaneous panniculitis. (A, B) Ultrasound of the right dorsal foot demonstrates innumerable subcutaneous nodules of echogenic fat pad in keeping with panniculitis. (C) Ultrasound of the right ankle demonstrates fluid in the tendon sheaths and increased vascularity consistent with tenosynovitis of the extensor hallucis longus and digitorum longus tendons.

Four weeks after the initial presentation, the patient developed a painful nodular rash on the lower limbs. Radiographs of the right foot revealed minor subcutaneous thickening, particularly at the posterior aspect of the right ankle this reflected the early changes of panniculitis. Ultrasound imaging of the left ankle revealed the presence of subcutaneous nodules containing echogenic fat consistent with inflammation. Furthermore, fluid was seen associated with tendon sheaths as well as thickened synovium, this is consistent with tenosynovitis (Figure 3A–C).

**Figure 4.**
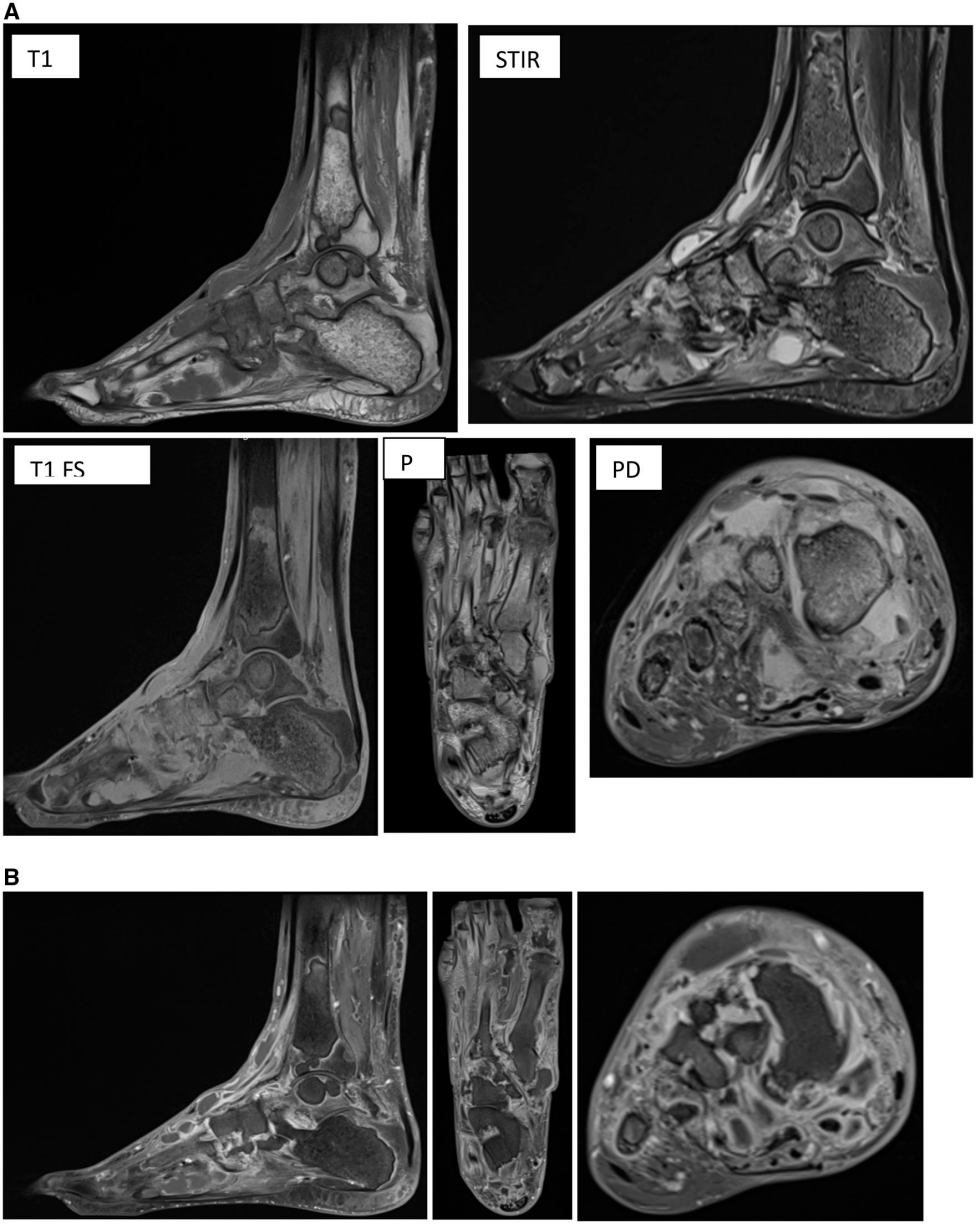
Multi-sequence MRI images of the right ankle demonstrating subcutaneous panniculitis and intramedullary fat necrosis. T1 FS post-gadolinium contrast in sagittal, long and coronal (left to right). (A) T1 fat saturated and STIR images demonstrate innumerable intramedullary lesions throughout the bones of the right lower limb. They have a serpiginous border with a double rim (on STIR sequence there is a hyperintense line paralleling the inner surface of the hypointense line within the marrow). Internally the lesions demonstrate T1 hypointensity with mild heterogenous T2 hyperintensity. (B) T1 post-gadolinium contrast images demonstrate mild peripheral contrast enhancement with adjacent bone oedema.

Subsequent MRI pre- and post-contrast of the right ankle, foot, and right knee confirmed the presence of these fluid signal intense subcutaneous lesions consistent with panniculitis, intramedullary lesions indicative of intramedullary fat necrosis. Increased synovial enhancement and thickening in keeping with polyarthritis (Figure 4A and B). These findings are in keeping with the ultrasound findings of soft tissue inflammation.

The consulted dermatology team recommended a tissue biopsy of the left thigh. The biopsy demonstrated lobular panniculitis with neutrophilic micro-abscesses in the subcutaneous fat. There was patchy fat necrosis with irregular sclerotic membrane with basophilic granular calcium deposition. This histological appearance was consistent with pancreatic panniculitis.

The patient’s treatment plan included the use of octreotide to inhibit the release of pancreatic exocrine enzymes, the application of compression bandages to alleviate swelling, and high-dose steroid therapy.

**Figure 5.**
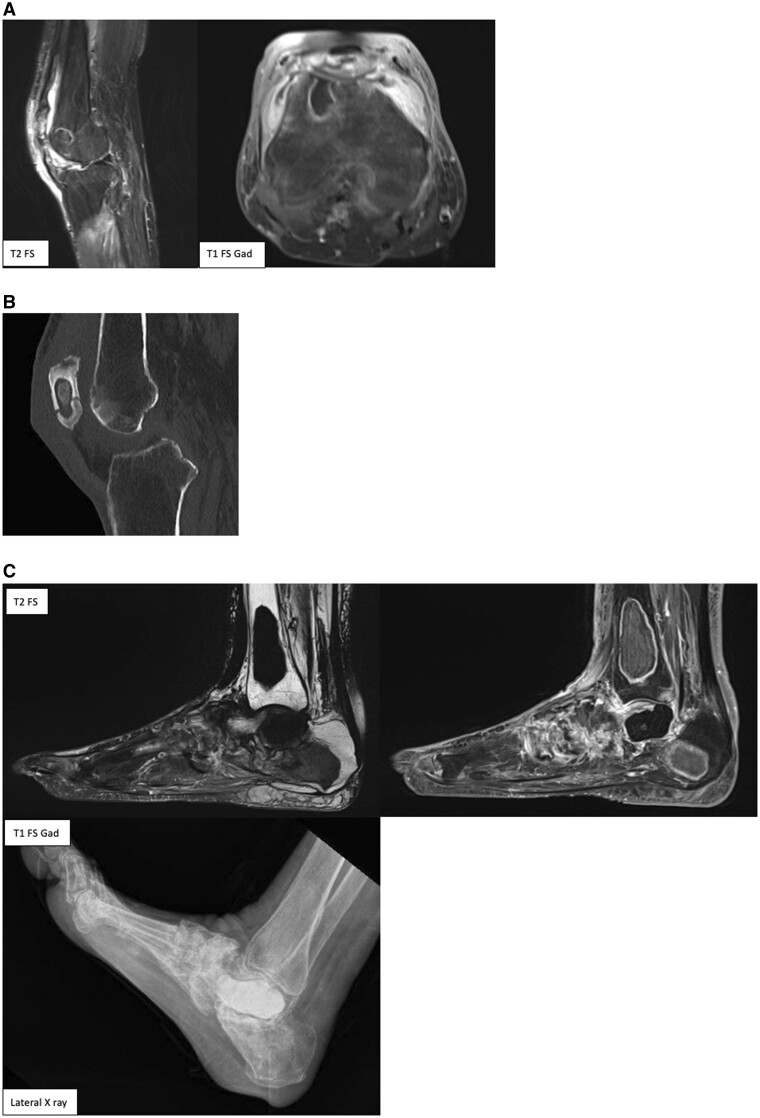
(A) Intramedullary fat necrosis with formation of cystic spaces in the distal femur and patella. Intramedullary lesions demonstrating T1- and T2-weighted low signal in the distal femur and patella. There is surrounding rim of enhancement. This is consistent with intramedullary fat necrosis. (B) Pathological fracture of the patella associated with intramedullary fat necrosis. Minimally displaced fracture line through the patella. There is a cystic space within the body of the patella which contains subtle calcification. (C) Intramedullary fat necrosis with cystic spaces and bone cement. Multiple lesions demonstrating low signal in T1- and T2-weighted images. There is partial collapse of the talus, which is filled with bone cement with very low signal T1 and T2 signal, this is seen with increased attenuation on radiograph.

The diffuse intramedullary fat necrosis created cavities in the distal right femur, patella, and left ankle (Figure 5B). This resulted in a pathological fracture of the right patella (Figure 5A) as well as collapse of the left talar dome. These cystic spaces were filled with bone cement to prevent further collapse, this appears as very low signal on T1- and T2-weighted MRI images and increased density on radiographs (Figure 5C).

The patient’s clinical course was further complicated due to the development of multifocal Candida septic arthritis, affecting the right knee, right ankle, and multiple metatarsal bones. Blood cultures, arthroscopic and open washout of the right knee identified *C albicans* and Stenotrophonmonas as the causative pathogens. Notably, the patient had a normal neutrophil count and tested negative for HIV in serology. The patient underwent debridement of the right patella and femur. The patient was received a further 3-month course of Fluconazole and Sulfamethoxazole.

The distinctive clinical progression observed in this patient highlights the uncommon complications associated with post-ERCP pancreatitis. As demonstrated in this case, the PPPS typically manifests within a period of 3-6 weeks following the peak of clinical pancreatitis. Moreover, it's essential to acknowledge that in some instances, osteomyelitis may occur following PPPS in the affected bones. In this case, the development of multifocal osteomyelitis may be attributed to multiple factors, including considerations related to bacteraemia, fungaemia, and PPPS.

## Discussion

Intra-abdominal complications following pancreatitis, such as pancreatic fluid collections, necrotizing pancreatitis, vascular complications, and fistula formation, are widely recognized. However, the lesser-known aspect of pancreatitis concerns extra-pancreatic complications, such as pancreatitis, polyarthritis, and panniculitis.

This case serves to emphasize post-procedural complications related to pancreas can also lead to PPPS.

Furthermore, aside from acute or chronic pancreatitis, pancreatic cancer, or pseudocysts may also be associated with PPPS.

When a painful nodular rash or arthralgia emerges after an episode of pancreatitis, it should trigger consideration of the possibility of pancreatitis, polyarthritis, and panniculitis syndrome (PPPS). This awareness can help prevent delayed diagnosis or misdiagnosis.^3^

The most accepted hypothesis for this condition is associated with the release of pancreatic enzymes, particularly lipase and various proteases, into the bloodstream. This process results in fat necrosis and the creation of fat emboli, subsequently causing inflammation in adipose tissues like periarticular bone marrow and subcutaneous fat. Additionally, other manifestations encompass synovial effusion, joint inflammation, and bone infarction.^1^

Some authors believe that the arthritis linked to pancreatic disease stems from elevated concentrations of free fatty acids in specific areas. In this scenario, lipolytic pancreatic enzymes adhere to the membranes of adipose cells, particularly those in periarticular or bone marrow tissues, triggering the hydrolysis of triglycerides into free fatty acids. Subsequently, these free fatty acids may be discharged into the joint, leading to the onset of acute arthritis.^4^

The primary risk factors frequently associated with this condition include male gender, occurrence typically in the 4th to 7th decades of life, a history of alcohol abuse, or a history of acute/chronic pancreatitis.^5^

Typically, the symptoms tend to be transient, although in a small number of cases, they may progress to a chronic state or become further complicated, as demonstrated in this specific case, where osteomyelitis emerged as a complication.^3^ The knees, ankles, wrists, and metacarpals are the most commonly affected sites.^6^

Osteoarticular involvement can lead to the development of synovial thickening/effusion, arthritic changes, osteolytic lesions and moth-eaten bone destruction, and serositis potentially resulting in permanent destruction and causing significant morbidity. Within the context of PPPS syndrome, arthritis can stem from either periarticular fat necrosis or the direct spread of subcutaneous fat necrosis into the neighbouring joint space.^7^

Intramedullary fat necrosis may occur as an isolated phenomenon or in combination with subcutaneous fat necrosis as in our case. This may present as moth-eaten bone destruction, periostitis and formation of cystic spaces. In our case, it is further complicated by partial collapse of the talar dome. MRI appearance is as seen in our case which comprises of T1-weighted low signal lesion and T2-weighted low signal lesion surrounded by a region of increased medullary signal.^5^

Bone infarcts on MRI display characteristic features, including a peripheral serpiginous pattern with low T1 signal and enhanced rim after contrast. In addition, they exhibit an ill-defined high signal intensity on T2 images, typically with a double-line appearance, consisting of a hypointense outer ring and a hyperintense inner ring.^8^

Subcutaneous nodules, which can either be painful or painless, often manifest as oedematous and red/bluish in colour, typically appearing on the lower extremities. In some instances, these nodules may ulcerate and discharge a thick, creamy substance, which is sterile. Pathological examination reveals septal necrotizing panniculitis with the infiltration of neutrophils.^9^

The occurrence of osteomyelitis following bone infarcts is uncommon. It is hypothesized that these infarcts essentially serve as “giant sequestra” that may harbour infectious microorganisms. In order for an infection to develop, certain conditions must be met, including vascular stasis and an environment conducive to bacterial growth. Regions affected by marrow infarcts may provide such a suitable medullary culture medium.^10^

## Learning points

- Highlights the rare complication of post-procedural pancreatitis: pancreatitis, panniculitis, and polyarthritis syndrome.
- Painful nodular rash and arthralgia are early clinical signs and symptoms.
- The development of subcutaneous nodules represents necrotizing panniculitis, this can be diagnosed on ultrasound in the first instance and confirmed on MRI.
- Intramedullary fat necrosis and bone infarcts are further complications which may be difficult to detect on radiograph and CT, these are more reliably diagnosed on MRI.
